# Correlates of COVID-19 vaccine uptake among the forcibly displaced: evidence from Libya

**DOI:** 10.1186/s13690-024-01306-4

**Published:** 2024-05-13

**Authors:** Meshack Achore

**Affiliations:** Department of Population Health, 220 Hofstra University, 101 Hofstra Dome, Hempstead, NY 11549-2200 USA

**Keywords:** Forcibly displaced, Displacement, COVID-19, Vaccine uptake, Libya

## Abstract

**Background:**

Vaccine hesitancy and refusal can hinder the control of infectious diseases such as coronavirus disease 2019 (COVID-19). Although forcibly displaced individuals are at high risk of contracting COVID-19, evidence shows that they are less likely to accept the COVID-19 vaccine. Given their predicament, the factors influencing vaccine uptake in the general population might differ vastly from those in displaced populations. Given the limited evidence on vaccine uptake from humanitarian settings, the current study examined the determinants of COVID-19 vaccine uptake among the forcibly displaced in Libya.

**Methods:**

Data were extracted from the World Bank/United Nations High Commissioner for Refugees (UNHCR) microdata repository. Data were collected between April and July 2021 after the rollout of the first dose of the COVID-19 vaccine in Libya. Percentages, means, and standard deviations were used to quantify the distribution of the sample population. Logistic regression models were employed to identify factors influencing COVID-19 vaccine uptake.

**Results:**

Odds ratios (ORs) with *p* values are used to present the regression analysis results. The study revealed that people unaffected by COVID-19 were less likely (OR = .71, 95%CI = 0.67–0.89) to accept the vaccine. Similarly, individuals with access to free COVID-19 vaccines were more likely to be vaccinated than those without free vaccines (OR = 38, 95%CI = 0.19–0.28). Finally, the results indicated that individuals were six times more likely to be vaccinated at mass vaccination sites ((OR = 6.31, 95%CI = 5.46- 7.94) and 1.92 times more likely to be vaccinated at local health centers (OR = 1.92, 95%CI = 0.1.72–3.11) than they were at hospitals and distant health facilities.

**Conclusion:**

Implementing comprehensive mass vaccination venues, public education initiatives, and awareness campaigns regarding the importance of vaccination can decrease vaccine hesitancy among the forcibly displaced.

**Table:** 

Text box 1. Contributions to the literature
1) Discussions surrounding COVID-19 have focused mainly on the spread and effectiveness of preventive measures such as hand hygiene and social distancing.
2) Although there is a body of literature on vaccine uptake, evidence on COVID-19-related vaccine uptake among the forcibly displaced population is limited.
3) This study contributes to the vaccine literature by identifying factors influencing COVID-19 vaccine uptake among people forcibly displaced in Libya.
4) Host country governments and organizations such as the United Nations and the World Bank can use this evidence to program and curate targeted interventions for people in humanitarian settings against future pandemics and epidemics.

## Background

The number of forcibly displaced populations is increasing globally. Estimates from the United Nations High Commissioner for Refugees (UNHCR) indicate that there are more than 60 million internally displaced people and 25 million refugees globally [39], https://www.unhcr.org/about-unhcr/who-we-are/figures-glance). Most (76%) dwell in low- and middle-income countries (LMICs), primarily in informal settlements, commonly known as refugee camps. Crowded camps undermine public health efforts to track and prevent the spread of infectious diseases, increasing vulnerability to outbreaks such as the coronavirus disease 2019 (COVID-19) caused by SARS-CoV-2. Reports from the World Health Organization (WHO) indicate that 25 million refugees live in overcrowded camps in host countries with inadequate healthcare facilities (http://www.who.int/health-topics/refugee-and-migrant-health#tab), further limiting their access to healthcare. Furthermore, the lack of access to basic amenities such as water, sanitation, and hygiene (WASH) further exacerbates the health challenges of the forcibly displaced [12, 18]. Given the limited access to healthcare resources and treatment services in host countries, preemptive measures such as vaccinations are imperative for slowing and preventing the spread of infectious diseases such as COVID-19.

There is no doubt that the COVID-19 vaccine has contributed significantly to saving lives through a reduction in the spread of the virus, hospitalizations, and mortality [19, 24, 41, 42]. Nevertheless, access to vaccines remains unequal, disproportionately affecting displaced populations due to factors such as distribution and the cost of production challenges [7]. This unequal distribution has resulted in low vaccination rates [7] and high mortality rates [44], https://covid19.who.int/) in LMICs, where forcibly displaced persons (e.g., refugees) reside. According to WHO reports, only 36.1% of inhabitants of LMICs received at least one dose of vaccine in March 2023, with the forcibly displaced population accounting for less than 2% of this population [44]. Factors attributed to the low vaccination rate among people forcibly displaced include immigration status, language barriers, and skepticism about vaccine effectiveness [1, 13].

COVID-19 vaccines, including Pfizer BioNtech or AstraZeneca (Vaxzevria), are generally robust for treating early strains of COVID-19, effectively reducing hospital admission rates, deaths, and illnesses [6, 32, 45]. However, subsequent waves brought new variants, such as Delta and Omicron, posing new challenges, notably a decrease in vaccine effectiveness attributed to diminished vaccine-neutralizing activity. Despite this, research indicates that while vaccines have reduced efficacy against specific variances in preventing transmission, they are still effective in protecting against catastrophic consequences [28, 36]. Introducing booster doses has proven crucial for enhancing immunity and improving vaccine efficacy, especially for emerging variants [8, 31]. The delta wave showed a notable decline in vaccine efficacy, with correlated spikes in mortality rates in Libya [3]. However, introducing the booster vaccine during the Omicron wave resulted in a positive shift. The booster vaccine provided another layer of protection, leading to a general decline in hospitalization and mortality rates [3].

### COVID-19 vaccine uptake

Effective vaccine rollout depends on availability, public acceptance, and adherence. Various studies [2, 7, 10] underline this critical relationship. Initially, vaccine uptake increased in the first few months but remained stagnant and plateaued, mirroring the wave patterns. For instance, in Libya, uptake surged before the third wave but declined thereafter [3]. For the most part, vaccines are accepted due to their ability to prevent infections [7, 10, 19, 25]. However, concerns about their effectiveness and fears of side effects hinder their use [33]. Factors contributing to vaccine hesitancy, the unwillingness or refusal to accept a vaccine despite its availability, vary by region. In developed countries, concern about the safety of the COVID-19 vaccine predominates as the main reason for hesitancy [10, 30], while in developing countries, where most displaced people are situated, factors such as lower-case fatality rates, trust, and conspiracy beliefs are more prevalent [5, 37]. Additionally, age, gender, and the credibility of the information source influence hesitancy [9, 25, 41]. For instance, in their search for a link between socioeconomic class and gender in influencing vaccine acceptance using intersectionality theory, Morales et al. found that women were more reluctant to receive vaccinations than men. The interaction between gender and socioeconomic class complicates people's vaccine hesitancy [30]. Specifically, women who worked or lived in poverty were less likely to be vaccinated, while men's hesitancy was unaffected by poverty or work [30].

Displaced populations, particularly refugees, face heightened vulnerability during infectious disease outbreaks and subsequent economic shocks, underscoring the need for the global community to find innovative ways of protecting this vulnerable population. Thus, there is a need to assess and understand the factors that facilitate or hinder vaccine uptake among forcibly displaced individuals. Understanding their unique social circumstances and the challenges they encounter, such as limited access to healthcare services and language barriers, underscores the importance of exploring the correlates of COVID-19 vaccine uptake among forcibly displaced people. Additionally, despite Libya being home to many refugees and migrants, there is limited evidence on the determinants of vaccine uptake among the displaced population in the country. This paper thus contributes to the existing evidence by examining the determinants of COVID-19 vaccine uptake among forcibly displaced people using evidence from Libya. The findings from this study will advance our understanding of the factors that hinder vaccine uptake and how vaccine perceptions may shift during public health emergencies. In addition, the findings will help host nations and international organizations such as the UNCHR and the World Bank with their programming and curation of targeted interventions to protect the forcibly displaced from future epidemics and pandemics.

## Methods

### Data sources

The data for this study were derived from high-speed socioeconomic data collected from a panel of refugees and migrants in Libya. The survey sought to examine refugee and migrant populations over approximately 18 months. The goal of the survey was to assess the socioeconomic activities of refugees and migrants in Libya and, in so doing, increase interagency collaboration and programming to address the vulnerabilities of displaced populations in Libya. The survey is projected to be conducted in four rounds. However, at the time of this analysis, only one round of datasets was available. The survey captures information on household composition, supplemented with data on individual characteristics (age, sex, areas of origin, levels of education, occupation, language, ethnicity, housing tenure), migration routes and costs incurred, types of vulnerabilities, migration and movement motives, future intentions, living challenges, and COVID-19 knowledge and vaccinations.

The baseline survey (round 1) was conducted between April and July 2021, after the rollout of the second dose of the COVID-19 vaccine in Libya. Due to access restrictions in Libya, the survey was carried out using phone calls. A third-party service provider was surveyed via phone from their offices in Amman, Jordan. Overall, 1,448 migrant and 2,019 refugee households were interviewed by phone. Round-one data were used for this analysis.

### Data availability and ethical considerations

The data were stored in the publicly accessible repository of the UNCHR/World Bank. To access the data, individuals must complete a registration process exclusively provided for legitimate research. Consent forms were administered following the principles of human subject protection at the household and individual levels.

### Measures

#### Outcome of interest: vaccine uptake

The respondents were asked about their vaccination status. Specifically, participants were asked to indicate yes or no to the following question: "Have you received the COVID-19 vaccine?" A binary variable was constructed based on the response, with 0 representing vaccinated and 1 representing unvaccinated.

#### Covariates of interest

Explanatory measure of interest were selected following a literature review of characteristics previously identified as risk factors for the outcome variable of interest [17, 38]. I adjusted for these variables in Model 2 (see Table 2). The variables were classified into (1) demographic variables, including age, sex, employment status, marital status, and health insurance status, and (2) vaccination status, which captured whether participants had received the COVID-19 vaccine (e.g., whether they had heard about vaccine campaigns or information and where they received their vaccination).

### Statistical analysis

Using data from the round 1 survey, bivariate, multiple, and univariate logistic regression were used to identify household characteristics related to vaccine uptake. Percentages, proportions, means, and standard deviations were used to determine the household characteristics of our sample. Using bivariate analysis, Model 1 investigates the association between group membership and vaccination uptake. The net relationships between the dependent and independent variables were further examined using multivariate analysis while accounting for several demographic, socioeconomic, and geographic aspects, with the group—refugees and migrants—as the focal independent variable. All analyses were performed using STATA 18. Odds ratios (ORs) with p values are used to present the regression analysis results. An OR greater than 1 indicates the likelihood of the event (accepting the vaccine), while an OR less than 1 indicates a reduced odd of the event. Confidence Intervals (CI) and *p*-value of < 0.05 was used to determine the significance of the associations.

Before the analysis, the following logistic regression assumptions were tested: (1) categorical dependent variables, (2) independent observations, and (3) outliers. All potential explanatory variables, including confounding variables, were screened using univariable analysis. According to the univariate analysis, variables with significance alphas less than or equal to 0.05 were included in the multivariate regression model (model 3). In addition, Spearman's correlation was used to assess pairwise correlations among the independent variables, with |r|> 0.7 considered highly correlated. A cumulative distribution function of the chi-square test was also used to identify outliers for continuous variables. An outlier is defined as a value greater than 0.001. We also assessed confounders and retained them in the model by calculating the magnitude of confounding (MoC). If the MoC exceeded 10%, we considered the variable a confounder. Correlation coefficients were calculated for each predictor variable to test for multicollinearity. There is collinearity when the correlation between variables is greater than 0.8.

## Results

**Fig. 1:**
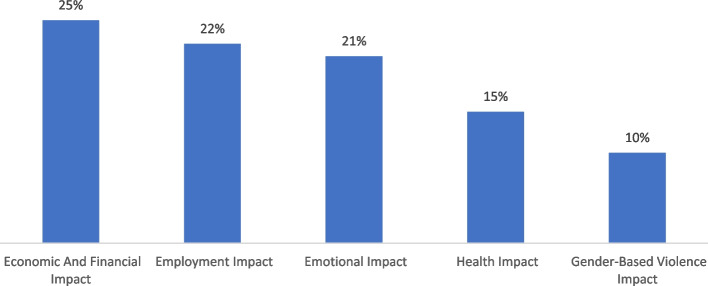
Impact of COVID-19 on households in Libya. Source: VSCM-S 2021

The socioeconomic characteristics of the study participants are presented in Table 1 below. COVID-19 has deleteriously impacted the forcibly displaced (see Fig. 1). Refugees account for 58.23% of the study sample; the rest are migrants. Approximately 52.29% of the respondents said they had not received a vaccine. Some of the reasons for refusing the vaccine included concerns about side effects (10.47%), friends and family advising me not to take the vaccine (7.26%), "My medical conditions prevented me (3.86%), mistrust in the vaccine (21.41%), and no vaccination campaign available (56.14%). Males made up 90.28% of the total sample. The rest of the demographic data are presented in the table.

**Table 1:** Characteristics of the study participants

Variables		Frequencies
Groups	Migrants	1,448 (41.77%)
	Refugees	2,019 (58.23%)
Gender	Females	336 (9.69%)
	Males	3,129 (90.28%)
Reasons for no vaccination	Concerned about side effects	271 (10.47%)
	Friends and family advised not to take	188 (7.26%)
	My medical conditions prevented me	100 (3.86%)
	medical personnel advised me not to	22 (0.85%)
	Mistrust in the vaccine	554 (21.41%)
	No vaccination campaign is available	1,453 (56.14%)
Free vaccine	Yes	2,666 (77.79%)
	No	761 (22.21%)
Covid-19 infected	Yes	993 (28.69%)
	No	2,468 (71.31%)
Employment status	Employed	1,838 (53.08%)
	Unemployed	1,625 (1,625%)
Marital status	Divorced	72 (2.30%)
	Married	2,023 (64.69%)
	Single	924 (29.55%)
	Widow	108 (3.45%)
Age	< = 20	224 (6.46%)
	from 21–40	2,308 (66.57%)
	> 40	935 (26.97%)
Vaccine status	No	1,808 (52.39%)
	Yes	1,643 (47.61%)

Table 2 below presents a bivariate and multivariate logistic regression examining the determinants of vaccine acceptance among refugees and migrants. Model 1 evaluates the association between the forcibly displaced group (i.e., migrants or refugees) and vaccine uptake. The results indicate that compared to migrants, refugees are less likely (OR = 0.35, 95%*CI* = 0.21–0.62)) to accept the COVID-19 vaccine. Model 2, on the other hand, presents an adjusted logistic regression of the determinants of COVID-19 vaccine uptake among the two groups. According to the results, the odds of COVID-19 vaccine acceptance increase with age. For instance, albeit the difference was not significant, those between the ages of 20 and less were 1.36 times more likely to accept the vaccine. The odds increased for those older than 40, indicating they were two times more likely to get the vaccine (OR = 2.09, 95% CI = 1.23- 5.21). Our findings suggest that married women are more likely (OR = 1.24, 95% CI = 1. 09–1.46) to accept the vaccine than their unmarried counterparts.

**Table 2:** Binary and multivariate logistic regressions examining the determinants of vaccine acceptance among refugees and migrants

Variables		Model 1 OR (95% CI)	Model 2 OR (95% CI)
Groups	Migrants	ref	Ref
	Refugees	.35 (0.21–0.62)**	.32 (0.20–0.58)**
Age	< 20yrs		Ref
	20–40 yrs		1.36 (0.97–3.89)
	> 40 yrs		2.09 (1.23- 5.21)**
Household size			.97 (0.72–0.99)**
Gender	Male		Ref
	Female		1.16 (0.99–1.40)
Marital Status	Divorced		Ref
	Married		1.24 (1. 09–1.46)*
	Single		1.21 (0.82–1.36)
	Widow		1.19 (0.89–1.32)
Employment Status	Employed		Ref
	Unemployed		1.10 (0.96–1.26)
Infected with COVID-19	Yes		Ref
	No		.71 (0.67–0.89)**
Access to free vaccine	Yes		Ref
	No		.38 (0.19–0.28)**
Information source	Media outreach programs		Ref
	Doctors/nurses/pharmacists/chemist		.97 (0.78–1.16)
	Family and friends		1.46 (1.28–1.49)**
	Celebrities and social media influencers		1.73 (0.95–2.21)
	Local government authority orders		1.04 (0.87–1.33)
Location of vaccine	Hospital/clinic		Ref
	Mass vaccination site		6.31 (5.46- 7.94)**
	local health center		1.92 (0.1.72–3.11)**
Constant		1.67 (1.48–2.01)	1.27 (1.09–1.40)

*OR* Adjusted Odds Ratio, *CI* Confidence interval

^*^p value < 0.05

^**^p value: < 0.01

Regarding the COVID-19 virus, the study revealed that people who were not affected were less likely (OR = 0.71, 95%CI = 0.67–0.89) to receive the vaccine. Similarly, individuals with free COVID-19 vaccine access were more likely to be vaccinated than those without free access (OR = 0.38, 95%CI = 0.19–0.28). Finally, the results indicated that individuals were six times more likely to be vaccinated at mass vaccination sites (OR = 6.31, 95%CI = 5.46- 7.94) and 1.92 times more likely to be vaccinated at local health centers (OR = 1.92, 95%CI = 0.1.72–3.11) than they were at hospitals and clinics.

**Fig. 2:**
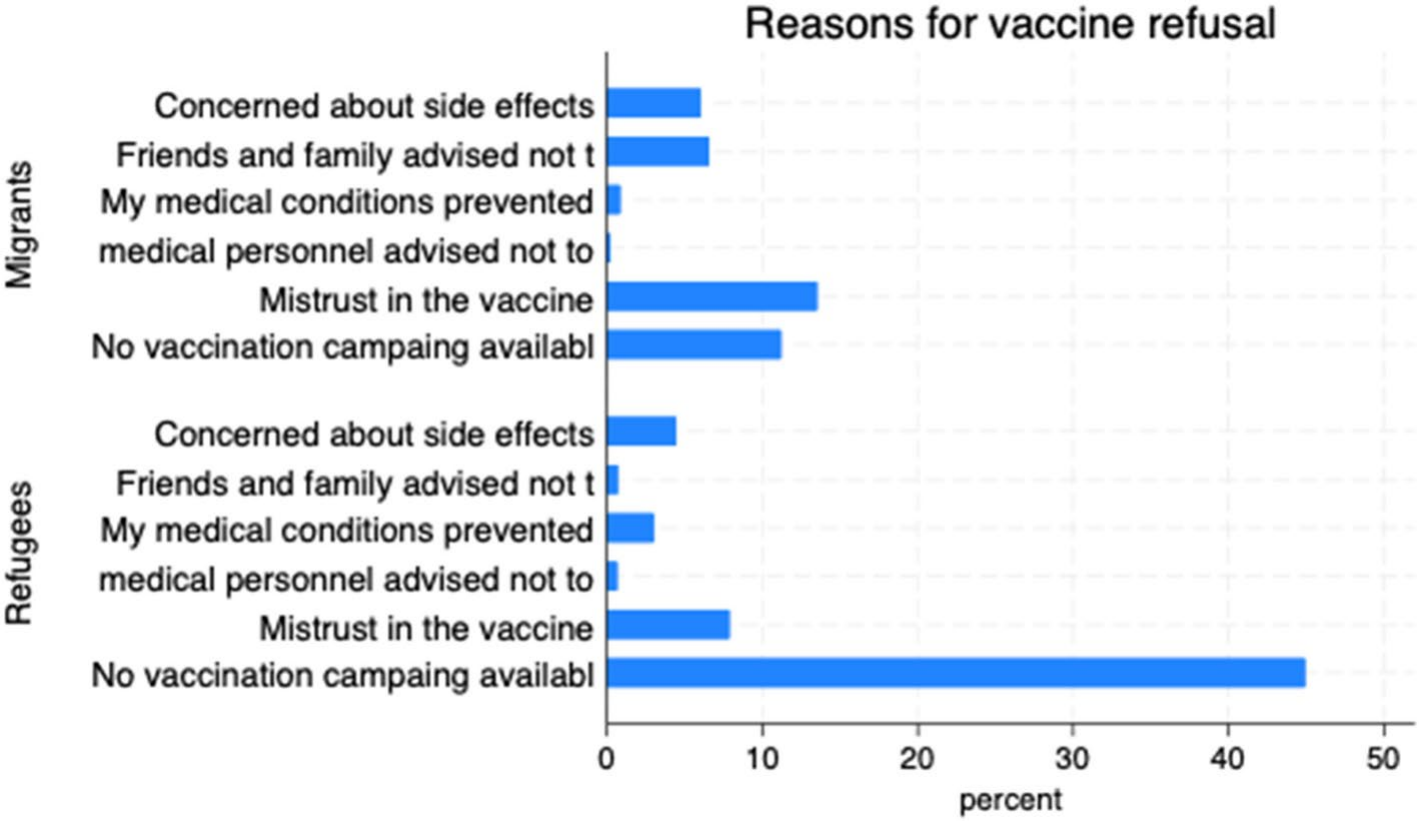
Reasons for COVID-19 refusal among migrants and refugees

Those who indicated they had not accepted the vaccine were further asked about the reasons for the refusal. For migrants, the first reason is mistrust of the vaccine (12%), followed by a lack of vaccination campaigns (11%). The opposite was found among refugees, with more than 45% indicating that no mass vaccine campaign was available. This means that they were not aware of the vaccine distribution. The second reason is mistrust of the vaccine (9%). The other reasons why people refused the vaccine are presented in Fig. 2 below.

## Discussion

Using data from the World Bank and the UNCHR data repositories, the current study examined the determinants of COVID-19 vaccine uptake among displaced populations in Libya. COVID-19 has had a deleterious impact on global health. Vaccines have become essential for combating the imminent threat of epidemics and pandemics such as Ebola and COVID-19. However, a segment of the global community hesitates to receive these vaccines despite their importance. Factors attributed to this hesitancy include cost, trust in healthcare providers, and sociodemographic factors such as age, employment, and educational attainment [9, 43]. A study by Hayward et al. [22] indicated that migrants are increasingly susceptible to COVID-19 and are disproportionately represented in confirmed virus cases. The available data suggest that undocumented migrants, migrant healthcare workers, and migrants residing in camps are particularly vulnerable to COVID-19 infection [15, 20].

The current study revealed that refugees are less likely to accept the COVID-19 vaccine than migrants. A plausible reason for this is choice; migrants choose to relocate, while refugees are forced to flee their homes for reasons stemming from persecution or conflict. Thus, most migrants might understand their host country's healthcare systems and language before relocating. These factors (i.e., language and healthcare access) are significant barriers for refugees. Most refugees neither understand the healthcare system nor speak the language of their host countries, limiting their access to preventive services such as vaccinations. Employment status is another plausible explanation for this difference. The employer mandated vaccinations at the height of the COVID-19 pandemic did not impact refugees because most host countries prevent refugees from working. On the other hand, in addition to fulfilling employer requirements, most migrants—in precarious employment—may be motivated to become vaccinated because their occupation predisposes them to the COVID-19 virus [22, 29]. However, contrary to the literature, our results indicate that employment status is not a determinant of vaccine uptake, meaning that other factors might explain why, compared to refugees, migrants are more likely to accept the COVID-19 vaccine. Future studies can further explore this relationship using qualitative methodologies.

Exploring factors contributing to hesitance toward accepting the COVID-19 vaccine is imperative for enhancing our understanding of the barriers to vaccine acceptance and the development of recommendations, particularly concerning communication strategies to overcome these barriers. The study identified six factors that hinder vaccine acceptance (see Fig. 2). A lack of trust in vaccines was a prominent factor influencing COVID-19 vaccine refusal among migrants. Although clinical trials and big data have demonstrated the safety and effectiveness of the vaccine in reducing transmission, hospitalization, and death [6, 32], most migrants expressed mistrust of the vaccine. Several factors contribute to this mistrust. First, although transient, adverse events such as headaches and injection site irritation made people feel uneasy and skeptical about the vaccine, and more adverse events such as myocarditis worsened these feelings. Second, research indicates that booster doses substantially increase antibody levels and improve defense against infections, particularly against new variants [8, 31],thus, booster doses are recommended for specific groups. However, introducing a third dose had the unintended consequence of creating doubts about the vaccine's efficacy, particularly for unvaccinated individuals. Questions such as "If the vaccines are effective, as argued, why do we need a third dose?" fueled the mistrust and conspiracy theories surrounding the COVID-19 vaccine. Another plausible reason for this mistrust is the effectiveness of nonpharmaceutical interventions (NPIs that prevent adverse COVID-19-related events across waves. For instance, although unsustainable and less cost-effective, in Hong Kong, NPIs such as travel restrictions, social distancing, and lockdowns effectively reduce hospitalizations and mortalities [45]. The lack of glaring adverse events showed that vaccines are not relevant for preventing transmission, and in most settings, they ignite conspiracy theories. This finding supports the burgeoning vaccine literature, showing that mistrust in vaccines, governments, healthcare systems, and healthcare providers decreases vaccine uptake [4]. For instance, a study by Allen et al. that examined the impact of medical mistrust on vaccine acceptance indicated a positive association between medical mistrust and vaccination status and intentions. This study further suggested that increasing COVID-19 vaccine acceptance will require public health to improve trust in vaccines and the medical system. Thus, strict surveillance systems are needed to monitor vaccine safety consistently and swiftly to evaluate any reported adverse events to maintain public trust [35].

The lack of vaccination campaigns was highlighted as the main reason for the lack of vaccination among refugees. The lack of vaccination campaigns and public health messaging to educate the populace on the significance of vaccination induces heightened public distrust, confusion, and skepticism. These findings are consistent with the findings of other scholars [11, 23, 40]. For instance, according to Johnson et al., educational campaigns about a disease-causing organism significantly improve people's attitudes toward vaccines. Chirico and Da Silva reported that the lack of credible, transparent, and science-based information and recommendations results in mistrust in governments and the public health system, consequently lowering the will to be vaccinated [11]. A study by Viswanath et al. reported a strong correlation between individuals' risk perceptions, namely, the severity of and susceptibility to COVID-19, and people's likelihood of receiving the vaccine [40]. Individuals who place their trust in news sites with conservative learning and have a diminished confidence level in the scientific community are the demographic least inclined to engage in vaccination for themselves and their children [40].

Choosing proper communication channels is imperative for public engagement through public health campaigns. The study found that individuals in humanitarian settings regard health information from family and friends as more credible than healthcare professionals (i.e., doctors, nurses, pharmacists), celebrities, and influencers. Thus, involving family members and friends who have received the vaccine is essential to encourage those who are hesitant about getting vaccinated. Public health campaigns promoting the safety and effectiveness of vaccinations, coupled with clear communication about the necessity of booster doses through family members with a history of vaccination, are crucial for enhancing acceptance and participation in COVID-19 vaccination programmes [16]. Providing information on vaccine safety, addressing concerns, and correcting misconceptions can enhance trust and confidence in vaccination initiatives.

One's health status is a significant predictor of vaccine uptake. According to the health belief model, behavior change has two components: 1) the desire to avoid or get well from illness and 2) the conviction that the behavior change will cure the illness or improve a condition. If a person does not see the immediate benefit of a behavior change, they are less likely to modify their behavior. Relatedly, an individual's perception of the severity and vulnerability of the COVID-19 virus influences their acceptability of the COVID-19 vaccine. Accordingly, we found that the odds of vaccination are lower among those not affected by the COVID-19 virus. This group has no sense of vulnerability or severity; hence, they do not see the immediate need to be vaccinated. Similar findings have been reported elsewhere [21, 40, 43]. For example, a study by Guthrie et al. [21] revealed that an increase in one's self-rated health is linked to greater odds of refusing the influenza vaccine because they deem it unnecessary. Individuals who believe that they have a robust immune system are less likely to accept the vaccine because they do not perceive any immediate threat or vulnerability to the COVID-19 virus.

We found that location is a significant determinant of vaccine uptake. The closer or more proximal the location of the vaccine site is, the lower the odds of vaccine hesitancy. Studies examining vaccine hesitancy have mainly concentrated on vaccine knowledge, trust, and risk perception. However, the distance to vaccination sites plays a significant role and is a noncoercive tool to improve vaccine uptake. Like our findings, a study by Mazar et al. (n.d.) examining the association between distance to vaccine sites and COVID-19 vaccine uptake revealed that location is an important yet overlooked determinant of vaccine acceptance. The study showed a consistently strong positive correlation between distance and vaccination rate. Displaced populations usually live in camps created by host countries and developmental agencies such as the World Bank, with most people never leaving these camps. Thus, they are unaware of the activities (e.g., vaccination campaigns, if any) beyond the confines of the camps, preventing them from receiving the vaccines. The study found that the odds of being vaccinated were significantly greater at mass vaccine sites erected near dwellings of the forcibly displaced. A plausible explanation for this finding is the proximity to the vaccination site. Therefore, it is imperative to consider the distance to vaccination sites in public health programming, as this distance plays a pivotal role in reducing vaccine hesitancy and addressing the ardent spread of infectious diseases among displaced populations already predisposed to infectious diseases due to the crowded nature of their camps.

Finally, the current study revealed that some sociodemographic factors are associated with vaccine uptake. For refugees and migrants, vaccination status is significantly associated with marital status and age. For example, compared to younger adults, older adults are more likely to be vaccinated. In their study, Robertson et al. [34] reported similar findings, suggesting that younger people are less likely than older people to receive vaccines. This is probably attributable to vulnerability, as older people are considered to be at high risk. Moreover, married couples were more likely to be vaccinated than single and divorced couples. This finding is consistent with a recent study by Liu et al. [27], who, in a study examining marital status and vaccine and COVID-19 vaccine uptake among older Americans, found that unmarried and divorced participants are less likely to accept the COVID-19 vaccine than their married counterparts. This finding is significant in the context of displacement because unmarried refugees and migrants are already dealing with their social and economic shocks alone, and the refusal to vaccinate exposes them to another layer of vulnerability.

### Limitations

Even with the valuable contributions made to the existing body of literature on migration and health, it is essential to acknowledge that the current study's findings may be subject to some limitations worth highlighting. First, this was a cross-sectional study; thus, causation could not be established. Second, this was secondary data and is associated with the limitations of secondary data, including the data not being collected for my specific research objective. Third, it is essential to recognize that individuals' self-reported vaccine hesitancy or acceptance may not necessarily align with their actual conduct. This discrepancy can be attributed to the dynamic nature of the COVID-19 pandemic, which necessitates a consideration of the time delay between the assessment of vaccine intention and the subsequent observation of behavior. Examining gender dynamics within the context of vaccine hesitancy is of utmost importance and warrants additional scholarly investigation, mainly through qualitative research methodologies.

## Conclusion

The present study demonstrated an association between migration status and vaccine uptake. Further scholarly exploration using qualitative research methodologies is needed to understand why people refuse or accept vaccines. This will aid policymakers in creating targeted interventions and programs to improve vaccine uptake among the forcibly displaced. In line with the findings of this study, interventions should target younger adults, unmarried couples, and households with few members, with a particular focus on creating, implementing, and enhancing initiatives that address vaccine hesitancy to mitigate the spread of infectious diseases. Implementing comprehensive mass vaccination venues, public education initiatives, and awareness campaigns regarding the importance of vaccination and herd immunity can decrease vaccine hesitancy among the forcibly displaced. In doing so, particularly in humanitarian settings, involving family and friends with vaccine history is imperative because they are more trusted. Furthermore, this study's findings suggest that bringing vaccination sites closer to the dwellings of forcibly displaced individuals may be an effective and noncoercive strategy for resolving vaccine hesitancy without requiring individuals to alter their beliefs.

Education efforts to enhance vaccine literacy and encourage evidence-based decision-making are crucial components of comprehensive vaccination plans [14]. Despite our findings indicating a lack of trust in healthcare workers, community health workers (CHWs) can contribute significantly to bridging this divide since they are trusted members of their communities. CHWs from camps can use their knowledge to inform camp residents about the benefits of vaccination, the dangers of COVID-19 infection, and the importance of herd immunity. When there are no CHWs from the camps, host country healthcare workers (HCWs) can act as intermediaries between trusted community members (i.e., family and friends) with vaccination histories and unvaccinated community members. They can provide resources and educational and communication skills to these groups, who, in turn, will educate unvaccinated community members.

Effective communication is essential for addressing concerns, dispelling conspiracy theories, and providing accurate information about the safety and efficacy of vaccines [26]. HCWs, as trusted sources of health information, play a pivotal role in delivering clear, evidence-based messaging to the public [16] by engaging in open dialog and addressing misconceptions. Healthcare providers may also engage in community activities to establish public trust before vaccine distribution. For example, healthcare workers can show exemplary behavior by taking vaccines in front of community members and sharing their experiences. In addition, HCWs play a pivotal role in controlling the spread of infectious diseases and protecting public health by empowering individuals to make educated decisions about vaccines and boosters by offering accurate information and personalized recommendations.

## Data Availability

No datasets were generated or analysed during the current study.
